# Genome-Wide Association Study of Arsenic Accumulation in Polished Rice

**DOI:** 10.3390/genes14122186

**Published:** 2023-12-07

**Authors:** Zheng Dong, Liang Guo, Xiaoxiang Li, Yongchao Li, Wenqiang Liu, Zuwu Chen, Licheng Liu, Zhixi Liu, Yujing Guo, Xiaowu Pan

**Affiliations:** 1 Hunan Rice Research Institute, Hunan Academy of Agricultural Sciences, Changsha 410125, China; 2 Key Laboratory of Indica Rice Genetics and Breeding in the Middle and Lower Reaches of Yangtze River Valley, Ministry of Agriculture, Changsha 410125, China

**Keywords:** arsenic, GWAS, MYB transcription factor, polished rice

## Abstract

The accumulation of arsenic (As) in rice poses a significant threat to food safety and human health. Breeding rice varieties with low As accumulation is an effective strategy for mitigating the health risks associated with arsenic-contaminated rice. However, the genetic mechanisms underlying As accumulation in rice grains remain incompletely understood. We evaluated the As accumulation capacity of 313 diverse rice accessions grown in As-contaminated soils with varying As concentrations. Six rice lines with low As accumulation were identified. Additionally, a genome-wide association studies (GWAS) analysis identified 5 QTLs significantly associated with As accumulation, with *qAs4* being detected in both of the experimental years. Expression analysis demonstrated that the expression of *LOC_Os04g50680*, which encodes an MYB transcription factor, was up-regulated in the low-As-accumulation accessions compared to the high-As-accumulation accessions after As treatment. Therefore, *LOC_Os04g50680* was selected as a candidate gene for *qAs4*. These findings provide insights for exploiting new functional genes associated with As accumulation and facilitating the development of low-As-accumulation rice varieties through marker-assisted breeding.

## 1. Introduction

The heavy metalloid contaminant arsenic (As) is prevalent and classified as a group I carcinogen, with no established safety threshold [1,2]. It easily enters the food chain through plants, posing a threat to all organisms including humans [3,4]. Arsenic pollution in farmland soil is increasingly exacerbated by ongoing industrial and agricultural activities. The primary factors contributing to the continuous rise in soil arsenic levels include mining and smelting activities, irrigation using groundwater contaminated with arsenic, and the utilization of arsenic-based fertilizers and pesticides [5,6,7,8,9]. The unique structure and paddy cultivation of rice enable it to accumulate high levels of arsenic, which distinguishes it from other cereal crops [10,11,12]. Arsenic contamination can adversely affect both the yield and quality of rice, as well as increasing the risk to humans of consuming arsenic-contaminated rice [13,14]. The presence of elevated levels of arsenic can lead to a range of acute and chronic health issues in humans, exerting detrimental effects on the cardiovascular, nervous, hematopoietic, renal, reproductive, and respiratory systems [15,16].

There are various forms of inorganic arsenic (AsIII and AsV) in the environment, and distinct transporters are responsible for the absorption and transportation of different arsenic species in rice plants [17]. The high-affinity phosphate (Pi) transporter proteins in rice play a vital part in the uptake and transport of AsV. The chemical analog of phosphate, AsV, competes with phosphate for root uptake through the same transport system but exhibits a higher preference for phosphate [18]. Several phosphate transporters (PHTs) and other phosphorous signaling regulatory proteins involved in AsV uptake have already been reported in rice, including OsPT1, OsPT4, OsPT8, OsPHF1, and OsPHR2 [19,20,21,22,23]. For instance, the overexpression of *OsPT8* significantly enhanced arsenic uptake in rice, whereas the *ospt8* mutant exhibited reduced arsenic uptake, thereby greatly enhancing plant tolerance to arsenic [20,22]. AsV can undergo rapid reduction to AsIII through enzymatic conversion catalyzed by arsenate reductases (AR), such as OsHAC1; 1/2 and OsHAC4 [17,24,25]. Unlike AsV, AsIII is taken up by root cells through aquaporin channels composed of Nodulin 26-like intrinsic proteins (NIPs), with the NIP subfamily predominating in AsIII uptake [26,27]. The OsNIP2;1 (Lsi1) protein in rice facilitates the uptake of AsIII, while the OsNIP2;2 (Lsi2) protein mediates the efflux of AsIII from root cells to the xylem in rice plants [27,28]. The presence of As in plant cells disrupts energy metabolism by replacing Pi in various metabolic processes [29,30].

At present, there are three available strategies to deal with arsenic pollution in rice production: using agronomic measures to reduce arsenic absorption by rice, screening rice genetic resources for low As accumulation, and creating rice with low arsenic accumulation through CRISPR/Cas-based gene editing [3]. Breeding rice varieties with low arsenic accumulation based on natural variation is one of the most effective ways to combat arsenic pollution in rice. However, the genetic mechanisms of As accumulation in rice are still unclear. Some series of QTL mapping have been reported using As-accumulation-related traits, including relative root length [31,32], relative seedling height [32], relative seedling dry weight [32], stem arsenic content [33], root arsenic content [33], leaf arsenic content [34], and grain arsenic content [33,34,35,36,37] under arsenic stress. Using a doubled haploid (DH) population of CJ06/TN1, Zhang [33] detected two major QTLs, controlling As accumulation on chromosomes 6 and 8, with additive effects of 0.081 and 0.092 mg/kg, respectively. Similarly, a major QTL, controlling As content in unmilled rice grain, was identified on chromosome 5 using the RIL population of Lemont/TeQing, and its Lemont allele reduced As content by 0.034 mg/kg [37].

To identify the rice genetic resources with low As accumulation and determine the responsible loci, we assessed the As accumulations in 313 accessions in various As-contaminated paddy fields. Six rice lines with low As accumulation were identified, and one of them maintained As levels below 0.2 mg/kg in 2020 and 2021. Genome-wide SNP discovery was carried out via site-specific amplified fragment sequencing (SLAF-Seq) [38], and, based on this, GWAS analysis was conducted to identify QTLs associated with arsenic content in rice. A total of 5 QTLs were detected in two experimental years, and they were distributed on chromosomes 1, 4, 5, and 12, respectively with only *qAs4* being detected in both years. Based on the results of gene expression analysis, we predicted *LOC_Os04g50680*, which encodes a MYB transcription factor protein, as the candidate gene for *qAS4*. The findings will contribute to the elucidation of the genetic mechanism underlying As accumulation and serve as a solid foundation for the development of low-As-accumulation rice varieties.

## 2. Materials and Methods

### 2.1. Field and Pot Experiments

A two-year field experiment was conducted in two arsenic-contaminated rice fields in Beishan, Hunan Province, China. A total of 338 rice accessions were collected from the Hunan Provincial Gene Bank, including landraces (148), bred varieties (82), and other domestic and international rice resources (108). The soil arsenic concentration was 12.8 mg/kg in 2020 and 17.3 mg/kg in 2021. Sowing dates were adjusted based on the growth duration of each accession to ensure synchronized heading. However, a few rice accessions were affected by high temperature, which led to a serious decrease in the setting rate. As a result, the number of arsenic accumulation samples was subsequently reduced to 313. After germination, the rice seeds were transferred to the rice seedling bed for cultivation. The seedlings were transferred to the experimental field at 25 days old. The accessions were each planted in two replications, and a randomized plot experiment was conducted. Each replication contained 16 rice plants planted in two rows, using within-row and between-row distances of 20 cm. Standard rice farming practices were followed for other field management tasks, including fertilizer application, disease control, and pest control. The seeds were harvested after maturity to determine the arsenic content in the rice grains. Hydroponic experiments were used to detect the expression level of candidate genes. The rice seeds were germinated for three days at 28 °C and 80% relative humidity (RH) in the dark. Seedlings with similar growth were selected and placed in hydroponic pots. The growth of the seedlings was continued for two days in the dark to allow root elongation, and they were then transferred to an IRRI nutrient solution. The solution was adjusted daily to pH 5.5 and renewed every 3 or 4 days, and the seedlings were germinated under conditions of 28 °C, 80% RH, and a 12 h light/12 h dark cycle.

### 2.2. Sampling and As Determination

The full and healthy rice grains were harvested 35 days after heading and subsequently subjected to a three-day drying process in an oven maintained at a temperature of 40 °C. The As concentration was determined according to the Chinese National Standard (GB 5009. 268-2016) [39]. The main focus of this study was to investigate the As accumulation in polished rice, which constitutes the primary edible component of rice. After the rice was crushed using a high-speed grinder, 0.5 g (Accuracy 0.001 g) of the sample was weighed into a microwave digestion tank, and 10 mL of nitric acid was added. The dissolution solution was dissolved in a microwave dissolver according to a heating program of 120 °C for 5 min, 150 °C for 10 min, and 190 °C for 20 min. The digested sample was removed and heated at 100 °C for 30 min, and then the volume was made up to 25 mL by adding ultra-pure water. After determining the sample volume, it and the blank solution were injected into the inductively coupled plasma mass spectrometer (ICP-MS) to measure the signal response values of the elements to be measured and the internal standard elements. The As concentration in the sample was calculated according to the standard curve.

### 2.3. SLAF-Seq Library Construction, Sequencing Screening, and SNP Calling Analysis

This part of library construction, sequencing data screening, and SNP analysis, was conducted according to our previous study [40]. Using the method proposed by Sun et al. [38], a SLAF library of each sequence was constructed and processed on a HiSeq sequencing 2500 system (Illumina, San Diego, CA, USA). This part of the process was performed by a bioinformatics corporation (Biomker, Beijing, China). Since most of our rice material belonged to the indica subspecies, the MEM algorithm using Burrows–Wheeler Aligner (version 0.7.10) software [41] was used to align the paired-end reads with the genome information of 93–11 (the pattern plant for indica rice, the URL is https://rice.genomics.org.cn/ accessed on 2 March 2022). After collating the results of the comparison with the 93–11 sequence, the data were filtered and screened for SNP calling using GATK (version 3.7) [42], Samtools (version 1.9) [43], and Plink software (version 1.9) [44].

### 2.4. Genome-Wide Association Study

DNA was extracted from the rice leaves at the tillering stage, and whole-genome sequencing was performed on 338 rice leaves using a simplified genome sequencing technique. Of samples, 313 were used for genome-wide association analysis of the arsenic content in rice based on a mixed linear model (MLM). The GEMMA software toolkit (version 0.98.5) was utilized to conduct association mapping in accordance with the methodology proposed by Zhou and Stephens [45]. To account for population structure, both the PCA matrix and the kinship matrix were included as covariates in this study. Significantly associated SNPs were identified using a threshold of *p* values ≤ 0.0001. The SNP with the lowest *p* value within a locus was designated as the lead SNP. The allele that contributed to the reduction in As content was considered the favorable allele, while the allele that increased As accumulation was considered the unfavorable allele.

### 2.5. Candidate Gene Prediction and Detection

The reference sequences of a 200 Kb window around *qAs4* were downloaded for gene annotation [46]. The *Indica* rice *93–11* was utilized as the background material for investigating the expression of *LOC_Os04g50680* in various tissues of the rice. For gene expression analysis, the rice seedlings were grown to a two-leaf stage and then sampled after 2 days of treatment with 25 μM Na_2_HAsO_4_.7H_2_O (Sigma, Saint Louis, MO, USA). The total RNA was extracted using Trizol and utilized for cDNA synthesis with a reverse transcription (RT) kit. Quantitative PCR (qPCR) analysis was conducted with a LightCycler 96 qPCR instrument (Roche, Rotkreuz, Switzerland) using a SYBR qPCR Mix kit. RNA extraction, RT, and qPCR kits were sourced from Vazyme (Nanjing, China). The qPCR primers for *LOC_Os04g50680* were 5′-GCATTGGTGCCTGAAACTTATC-3′ and 5′-TCCGAACTCCGACAACAGTC-3′, while the primers for the reference gene *Actin* were 5′-CATTGGTGCTGAGCGTTTCC-3′ and 5′-AGAAACAAGCAGGAGGACGG-3′.

## 3. Results

### 3.1. Variation in As Accumulation in Polished Rice

**Figure 1:**
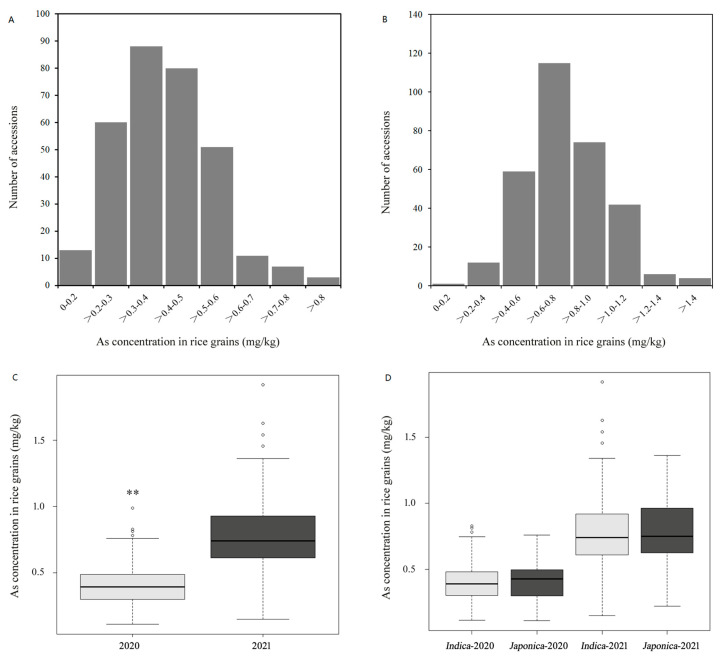
The As concentration in polished rice: distribution and comparative boxplots analysis. As concentration distribution in 2020 (**A**) and 2021 (**B**); (**C**) comparative boxplot analysis of As concentration in 2020 and 2021; and (**D**) comparative boxplot analysis of As concentration in *Indica* and *Japonica* rice subgroups in 2020 and 2021. ** indicates *p* < 0.01 in the Student’s *t*-test analysis.

The As content in the polished rice samples harvested from fields contaminated with arsenic was subjected to analysis using ICP-MS. The As accumulation showed significant variation among diverse rice accessions in 2020 and 2021 (Supplementary Table S1). The soil As concentration was 12.8 mg/kg in 2020 and 17.3 mg/kg in 2021. In 2020, the As concentrations in polished rice ranged from 0.11 mg/kg to 0.99 mg/kg, with an average of 0.40 mg/kg and a median of 0.39 mg/kg (Figure 1A). The As concentrations among 313 lines for two years were positively correlated with each other, but the coefficients were low (r = 0.4864, *p* < 0.001). The As concentrations of 13 rice lines were found to be below the permissible limit of 0.2 mg/kg, as stipulated by China’s national standard for food hygiene (GB 2762-2022) [47]. The arsenic accumulation data in 2021 ranged from 0.15 mg/kg to 1.92 mg/kg, the mean and median arsenic concentrations being 0.77 mg/kg and 0.74 mg/kg (Figure 1B), and just one line showed As concentrations below 0.2 mg/kg. Overall, the As accumulation in rice in 2021 was significantly higher than that in 2020, suggesting that the soil As concentration was an important factor affecting As accumulation in the rice (Figure 1C). Variance between the two years was highly significant (*p* < 0.0001), contributing 39.4% to the total phenotypic variance of As accumulation (Supplementary Table S2). Despite the significant differences between the two years, there were still six rice lines with consistently low levels of As accumulation (below 0.3 mg/kg) in both years (Table 1). Among them, the W176 (Chenwan-3) line exhibited the lowest accumulation of As (0.15 mg/kg) in 2021 and also demonstrated remarkably low levels of As (0.13 mg/kg) in 2020. Since this rice panel contained two subspecies, *Indica* and *Japonica*, we compared the As accumulations between them and found no significant difference between these two subspecies in either 2020 or 2021 (Figure 1D). These results indicate that the genetic diversity of varieties, rather than of subspecies, determines As accumulation in rice.

### 3.2. GWAS Analysis of QTLs Associated with As Accumulation

**Figure 2:**
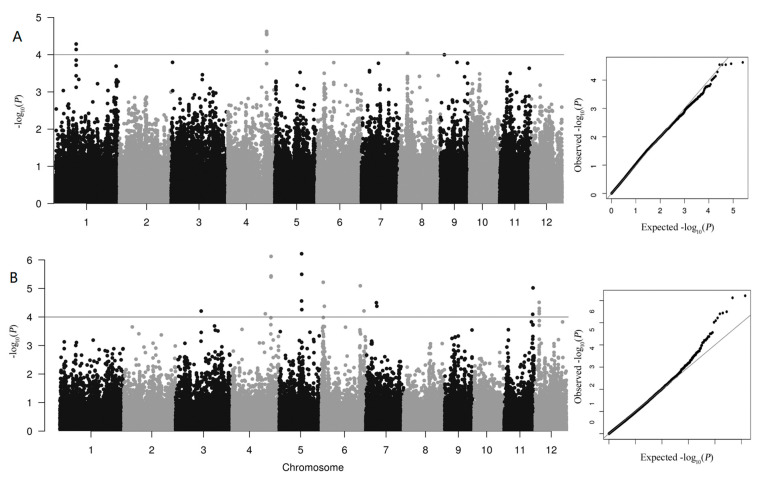
Manhattan plots and quantile–quantile (Q-Q) plot of GWAS analysis for As accumulation in polished rice in 2020 (**A**) and 2021 (**B**). The horizontal line in the Manhattan plots represents the threshold for the *p* value.

In order to investigate the genetic basis of As accumulation in polished rice, we conducted a GWAS analysis to identify the SNP loci associated with As accumulation in this natural population of 313 rice lines. A mixed linear model (MLM) was employed, incorporating the kinship matrix and PC matrix as covariates, thus reducing the interference of population structure in the GWAS analysis results. A total of 30 SNPs were identified as having significant associations with As accumulation in the two experimental years (Supplementary Table S3, Figure 2), supported by well-fitted quantile–quantile (Q-Q) plots (Figure 2). In line with the approach of a previous study [46], a region was considered as one QTL when more than two significant SNPs (*p* < 0.0001) were identified within a 200 Kb window. Based on this criterion, five QTLs were detected in this analysis, which were distributed on chromosomes 1, 4, 5, and 12, respectively. Comparing the QTLs identified in these two consecutive years, we consistently detected *qAs4*, implying that environmental factors may have significantly impacted the GWAS analysis results regarding As accumulation.

### 3.3. Candidate Gene Analysis for As Accumulation

**Figure 3:**
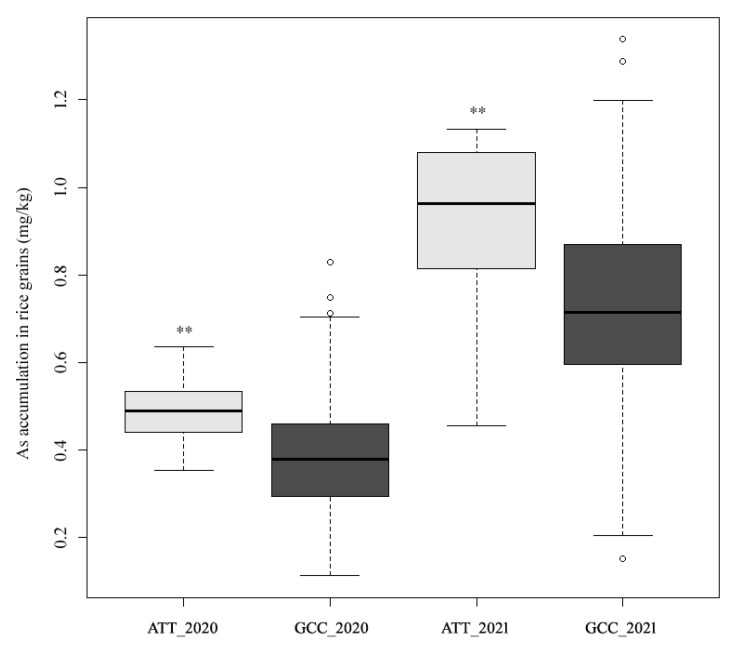
Distribution of As accumulation in polished rice between the favorable group GCC and unfavorable group ATT. ** indicates *p* < 0.01 in the Student’s *t*-test analysis.

It was found that six consecutive SNPs were significantly associated with As accumulation around the interval of *qAs4* (Supplementary Table S3), among which three SNPs (*rs4_28644370*, *rs4_28589826*, and *rs4_28654066*) were identified in both years. Based on the alleles of these three SNPs, the samples were classified into two groups: a group with a favorable allele GCC (referred to as group GCC) and a group with an unfavorable allele ATT (referred to as group ATT). As accumulation in group GCC was significantly lower than that in group ATT. In 2020, group GCC exhibited an average concentration of 0.38 mg/kg and a median concentration of 0.37 mg/kg, while group ATT showed an average concentration of 0.52 mg/kg and a median concentration of 0.50 mg/kg. In 2021, group GCC displayed an average concentration of 0.75 mg/kg and a median concentration of 0.71 mg/kg, whereas group ATT demonstrated a mean and median concentration of 0.98 mg/kg (Figure 3).

**Figure 4:**
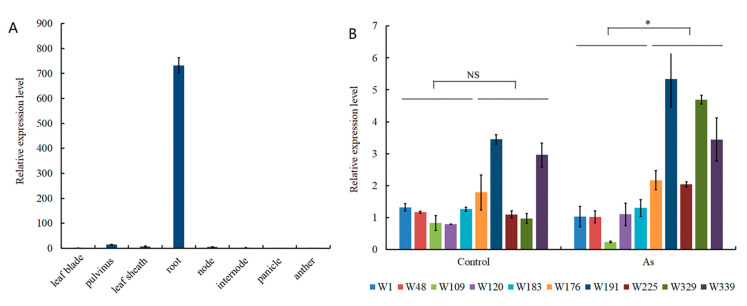
Expression analysis of the candidate gene *LOC_Os04g50680*. (**A**) Expression analysis of *LOC_Os04g50680* in tissues; (**B**) expression analysis of *LOC_Os04g50680* between high-As-accumulation lines (W1, W48, W109, W120, W183) and low-As-accumulation lines (W176, W191, W225, W329, W339) in no-AS and As (25 μM Na_2_HAsO_4_·7H_2_O) treatment. NS means nonsignificance. Significance was determined to be at a level of * *p* < 0.05.

In the annotation based on the reference genome, we identified 73 genes within the 200 Kb window of the lead SNP (*rs4_28644370*). One of the genes, *LOC_Os04g50680*, encodes the MYB transcription factor protein, whose expression has been reported to be induced by As stress [48]. Expression analysis showed that *LOC_Os04g50680* was expressed almost exclusively in the root and very weakly in other tissues and organs such as the leaf blade, pulvinus, node, and anther (Figure 4A). According to the genotype of *qAs4*, we selected 10 lines with contrasting As accumulations, including five high-As-accumulation lines (ATT) and five low-As-accumulation lines (GCC). Under normal growth conditions, there were no significant differences in the expression of *LOC_Os04g50680* between the low-As-accumulation accessions and the high-As-accumulation accessions (Figure 4B). However, the expressions in low-As-accumulation accessions were up-regulated after As treatment, and their expressions were significantly higher than those in high-As-accumulation accessions. The results indicate that *LOC_Os04g50680* may be a promising candidate gene for *qAs4*. The findings also suggest that the variation in SNPs in groups ATT and GCC may impact the up-regulation of transcription levels of *LOC_Os04g50680* following arsenic treatment, thereby influencing arsenic accumulation in rice.

## 4. Discussion

Natural genetic variation in rice not only plays a crucial role in breeding but also provides valuable insights into the genetic mechanisms underlying complex traits. The accumulation of As in rice exhibits significant variation among different rice accessions, indicating the potential for breeding rice varieties with low levels of As accumulation. In order to accurately assess the As accumulation capacity of different rice genetic resources, we cultivated 313 rice lines in paddy fields over two consecutive years. Rice grown in soils with higher As concentrations in 2021 accumulated more As than rice grown in soils with lower As concentration in 2020. These results were consistent with previous studies [3,49], indicating that the accumulation of As in grains was significantly influenced by the concentration of As in the soil. The genome-wide association mapping revealed a large number of loci associated with variations in mineral accumulation in rice grains across five environmental experiments [50]. The As accumulation in this experiment varied significantly, not only across environments but also across the years in the same area [50]. In our previous study, we found that cadmium (Cd) accumulation differed significantly between the *Indica* and *Japonica* groups within this rice population [40]. However, there was no significant difference in As accumulation between the *Indica* and *Japonica* groups in this study. The findings suggest that rice has distinct uptake and transport mechanisms for As and Cd. Unfortunately, the majority of the rice lines exhibited elevated levels of As accumulation exceeding 0.2 mg/kg throughout the two-year experimental period. Interestingly, we found a few rice lines with relatively low As accumulation in both years, mainly composed of landraces from Hunan province, China. Rice landraces have undergone extensive natural and artificial selection over a long period, resulting in remarkable genetic diversity and adaptability to different environmental conditions [51]. Our findings suggest that these landraces have the potential to serve as excellent genetic resources for developing rice varieties with low As accumulation.

The difficulty in identifying loci linked to complex characteristics in rice arises from the differentiation of populations [52]. To address this challenge, the MLM model has been widely adopted for its efficacy in controlling confounders and reducing false positives [53]. The variance in As content between the two years in this study was found to contribute 39.4% to the total phenotypic variance. This finding aligns with the understanding that environmental factors can have a significant impact on phenotype evaluation in GWAS [54]. In all identified QTLs, *qAs4* was consistently detected at different soil arsenic concentrations over two years, suggesting that *qAs4* might be a dominant QTL controlling As accumulation in rice (Table 2). The remaining QTLs were detected in only one year, and it is hypothesized that the effects of these QTLs may be smaller than or more influenced by environmental factors.

The root is the most important organ for the uptake of heavy metals in rice plants. The high expression of *LOC_Os04g50680* in the roots suggests its potential involvement in As absorption. LOC_Os04g50680 is annotated as a MYB transcription factor and exhibits up-regulation in response to As treatment. MYB proteins encompass a diverse array of transcription factors in plants and have been replicated in plant responses to various biotic and abiotic stresses, including phosphate deprivation, low-temperature exposure, and salt and drought stress [55,56]. The R2R3 MYB transcription factor OsARM1 (Arsenite-Responsive MYB1) governs the expression of AsIII-associated transporter genes, such as *OsLsi1*, *OsLsi2*, and *OsLsi6* [57]. Knockout of *OsARM1* enhanced the tolerance of rice plants to AsIII, while the overexpression of *OsARM1* increased sensitivity to AsIII [57]. *MYB40*-overexpressing lines consistently had a reduced As uptake rate in *Arabidopsis*. *MYB40* plays a central role in arsenic resistance, and it influences the As/Pi uptake through repressing the expression of the Pi transporter gene *PHT1;1* [58]. In addition, the mutation of another MYB transcription factor gene, *OsPHR2*, reduced the uptake and translocation of As, while the overexpression of *OsPHR2* enhanced the abilities of As absorption and transportation [20]. Therefore, we predicted *LOC_Os04g50680* as a candidate for *qAS4*. The expressions of *LOC_Os04g50680* in low-As-accumulation accessions were higher than those in high-As-accumulation accessions, implying that this gene might be a negative regulator of As accumulation; however, the detailed regulatory mechanisms remain to be studied in the future.

## 5. Conclusions

We identified six rice lines out of 313 accessions that exhibiting low levels of arsenic accumulation when grown in soils contaminated with varying concentrations of arsenic. These lines hold potential as donors for future breeding programs aimed at developing low-As-accumulation rice varieties. During a two-year experimental period, GWAS analysis successfully identified five QTLs that exhibited an obvious correlation with As accumulation. Among these QTLs, only *qAs4* was consistently detected in both experimental years. This locus has not been reported previously and was considered to be a novel QTL. Through expression analysis under arsenic stress conditions, we identified *LOC_Os04g50680*. This encodes a MYB transcription factor as a candidate gene for *qAs4*. The expression of *LOC_Os04g50680* was found to be up-regulated in the rice lines with low As accumulation compared to those with high accumulation after As treatment, implying that this gene might be a negative regulator of As accumulation. Additionally, the expression of *LOC_Os04g50680* was found to be high in the roots, corresponding to the main site of arsenic uptake and transport. However, the detailed regulatory mechanisms remain to be studied in the future. The findings presented here offer valuable insights for further exploration of novel functional genes associated with As accumulation and for the development of rice varieties with reduced As accumulation through marker-assisted breeding.

## Figures and Tables

**Table 1:** List of some low-As-accumulation rice accessions.

Field ID	As-2020	As-2021	Accession Name	Type
W165	0.23 ± 0.0127	0.26 ± 0.0134 *	Baimidongzhan	Landrace
W176	0.13 ± 0.0106 *	0.15 ± 0.0374 *	Chenwan-3	Bred variety
W191	0.25 ± 0.0233	0.22 ± 0.0537 *	Feitianzao	Landrace
W329	0.12 ± 0.0092 *	0.20 ± 0.0276 *	Aizizhan	Landrace
W348	0.27 ± 0.0240	0.30 ± 0.0113	Hong-410	Landrace
W351	0.29 ± 0.0170	0.29 ± 0.0078 *	Jiayu-948	Bred variety

* indicates *p* < 0.05 in the Student’s *t*-test analysis.

**Table 2:** List of QTLs related to As accumulation detected by GWAS analysis.

QTLs	Chr.	Position of Lead SNP	allele0	allele1	*p*-Value
2020					
*qAs1*	1	15248892	G	A	5.16 × 10^−5^
*qAs4*	4	28589826	G	A	2.36 × 10^−5^
2021					
*qAs4*	4	28644370	C	T	7.52 × 10^−5^
*qAs5*	5	16538553	C	T	6.08 × 10^−7^
*qAs12*	12	2908161	A	G	3.06 × 10^−5^

## Data Availability

Data are contained within the article and supplementary materials.

## Supplementary Materials

The following supporting information can be downloaded at https://www.mdpi.com/article/10.3390/genes14122186/s1. Table S1: As accumulation in 313 rice accessions. Table S2: Analysis of variance in the phenotypic performance of As accumulation in polished rice. Table S3: The significant SNPs of As accumulation.
